# Deep brain stimulation of the anterior nucleus of the thalamus for drug-resistant epilepsy

**DOI:** 10.1007/s10143-017-0941-x

**Published:** 2018-01-06

**Authors:** Tim A. M. Bouwens van der Vlis, Olaf E. M. G. Schijns, Frédéric L. W. V. J. Schaper, Govert Hoogland, Pieter Kubben, Louis Wagner, Rob Rouhl, Yasin Temel, Linda Ackermans

**Affiliations:** 1 Department of Neurosurgery, Academic Center for Epileptology (ACE), Maastricht University Medical Center, Maastricht (MUMC), PO Box 5800, 6202 AZ Maastricht, The Netherlands; 2 European Graduate School of Neuroscience (Euron), Maastricht University, Maastricht, The Netherlands; 3 School for Mental Health and Neuroscience (MHeNS), Maastricht University, Maastricht, The Netherlands; 4 Department of Neurology, Academic Center for Epileptology (ACE), Kempenhaeghe, MUMC, Maastricht, The Netherlands; 5 Academic Center for Epileptology MUMC+ and Kempenhaeghe, Heeze, Maastricht, The Netherlands

**Keywords:** Deep brain stimulation, Epilepsy, Anterior nucleus of the thalamus, Mechanisms, Efficacy, Complications

## Abstract

Despite the use of first-choice anti-epileptic drugs and satisfactory seizure outcome rates after resective epilepsy surgery, a considerable percentage of patients do not become seizure free. ANT-DBS may provide for an alternative treatment option in these patients. This literature review discusses the rationale, mechanism of action, clinical efficacy, safety, and tolerability of ANT-DBS in drug-resistant epilepsy patients. A review using systematic methods of the available literature was performed using relevant databases including Medline, Embase, and the Cochrane Library pertaining to the different aspects ANT-DBS. ANT-DBS for drug-resistant epilepsy is a safe, effective and well-tolerated therapy, where a special emphasis must be given to monitoring and neuropsychological assessment of both depression and memory function. Three patterns of seizure control by ANT-DBS are recognized, of which a delayed stimulation effect may account for an improved long-term response rate. ANT-DBS remotely modulates neuronal network excitability through overriding pathological electrical activity, decrease neuronal cell loss, through immune response inhibition or modulation of neuronal energy metabolism. ANT-DBS is an efficacious treatment modality, even when curative procedures or lesser invasive neuromodulative techniques failed. When compared to VNS, ANT-DBS shows slightly superior treatment response, which urges for direct comparative trials. Based on the available evidence ANT-DBS and VNS therapies are currently both superior compared to non-invasive neuromodulation techniques such as t-VNS and rTMS. Additional in-vivo research is necessary in order to gain more insight into the mechanism of action of ANT-DBS in localization-related epilepsy which will allow for treatment optimization. Randomized clinical studies in search of the optimal target in well-defined epilepsy patient populations, will ultimately allow for optimal patient stratification when applying DBS for drug-resistant patients with epilepsy.

## Introduction

Epilepsy is a common chronic neurological disorder characterized by spontaneous recurrent seizures and affects around 70 million patients worldwide [58]. Of these patients, over 30% will suffer from persistent seizures despite (optimal) anti-epileptic drug (AED) regimens [37]. Drug-resistant epilepsy is defined as a failure of two adequate trials of AEDs that are appropriate for the person’s disease [36]. The pathogenesis of drug-resistant epilepsy is not completely understood. However, both biological mechanisms and environmental factors are known to contribute to the development of drug resistance [14]. Persistent epileptic seizures and long periods of incomplete seizure control have profound social, physical, and psychological consequences leading to a decline in quality of life and impose a financial burden [72]. Moreover, patients with epilepsy are at risk for sudden unexpected death (SUDEP) which is a prominent cause for the elevated mortality-ratioin chronic epilepsy. Its annual incidence ranges from 0 to 10 per 1000 in epilepsy surgery candidates [75]. Risk factors include frequent generalized tonic-clonic seizures, AED polytherapy and an early onset of drug-resistant epilepsy [25].

If the epileptogenic focus or network can be localized and if the benefits outweigh the risks, resective surgery is effective when compared to medication treatment alone [88, 89]. A meta-analysis estimated that 67% of epilepsy patients showing MRI abnormalities treated with surgery were seizure free at 1 year vs. only 55% in patients with absent MRI abnormalities [15, 73]. Patients who cannot benefit from curative, resective surgery, can be referred for neuromodulation therapy, e.g., vagal nerve stimulation (VNS) or deep brain stimulation (DBS). In the case of DBS, several anatomical targets have been identified for neuromodulation of drug-resistant intractable epileptic seizures including the centromedian nucleus (CM) of the thalamus, the hippocampus, and the anterior nucleus of the thalamus (ANT), the latter of which gained widespread attention after the publication of the SANTE trial, a large double-blind, randomized trial in 110 patients with localization-related epilepsy [16, 70].

Here, we present a review of DBS of the anterior nucleus of the thalamus in patients with drug-resistant epilepsy and discuss its rationale, clinical efficacy, safety, tolerability, and mechanism of action. Further, we will discuss future steps of identifying DBS as a third line treatment modality in drug-resistant epilepsy, within the spectrum of neuromodulation techniques.

## Methods

Literature for this review was identified searching Medline, Embase, and the Cochrane Library databases from the date of the first available article until September 2017. The following keywords were queried either individually or combined: deep brain stimulation, epilepsy, anterior nucleus of the thalamus, complications, and mechanism of action. The search was limited to studies published in English.

## Rationale

### ANT anatomy and function

The ANT is situated in the rostral end of the dorsal thalamus and is separated from the rest of the dorsal thalamus through a Y-shaped internal medullary lamina. The ANT consists of the anteroventral (AV), anterodorsal (AD), and anteromedial (AM) subnuclei. As part of the limbic circuit of Papez, the ANT receives input from the hippocampal subiculum either directly via the fornix or indirectly via the mammilothalamic tract from the mammillary bodies (MB). Other afferents to the ANT originate from the anterior and posterior cingulate cortex, retrosplenial cortex, and the inferior parietal lobule [12]. Many of these cortical connections are reciprocal. Its putative functions comprises the involvement in relay of visceral and emotional information to prefrontal areas (AM), the modulation of alertness and as a component of an ‘extended hippocampal system’ in different aspects of learning, episodic memory and in spatial navigation (AD). The majority of neurons in the AV subnucleus fire synchronous with the hippocampal theta frequency, which has been implicated in spatial cognition [30, 86].

### ANT and epilepsy

The recognition of the putative role of the ANT in epilepsy, emerged from several animal studies in the second half of the twentieth century. In a model of focal cortical epilepsy in Rhesus monkeys, ipsilateral lesions in the ANT led to a significant decrease in frequency and duration of seizure generalization [35]. Further, pharmacologically mediated inhibition of the ANT in guinea pigs with bilateral injection of the γ-aminobutyric acid (GABA) agonist muscimol showed suppression of high-voltage synchronous EEG activity and behavioral components of pentylenetetrazol (PTZ) induced seizures, in a dose-dependent manner [51]. Correspondingly, bilateral ANT injections of γ-vinyl-γ-aminobutyric acid (vigabatrin), a suicide inhibitor of the enzyme GABA-transaminase, produced significant protection against PTZ-induced tonic-clonic seizures in rats [49, 51]. The functional role of the ANT in PTZ-induced seizure propagation was therefore hypothesized to be a relay nucleus to mediate paroxysmal activity between its associated subcortical structures and cerebral cortex. This was supported by earlier findings that lesions in the mammillothalamic tract significantly attenuated EEG activity and lethal effects of PTZ. Furthermore, chronic stimulation or single shock of either the fornix, MB, mammilothalamic tract or the ANT, induced cortical EEG discharges including seizure like activity [20, 50]. The latter of which implicated involvement of the Papez circuit in seizure propagation.

Subsequently, it was reported that specific electrical stimulation of the MB resulted in a protective effect against seizures [52]. In agreement with neuroanatomical identified MB-ANT connections, bilateral high frequency stimulation at 100 Hz (300-500 μA) of the ANT in rats doubled the dosage of PTZ required to elicit clonic motor seizures, but did not alter the expression of low dose PTZ-induced cortical bursting. High frequency stimulation of the ANT leads to EEG desynchronization, rendering the cortex less susceptible to seizures [52, 53]. In contrary, low frequency stimulation with 8 Hz proved to be a proconvulsant stimulus, as it lowered the threshold for early EEG paroxysmal bursts [53]. Although these findings support the concept of ANT mediation of cortical-subcortical interactions in PTZ-induced seizures, the specific synaptic or membrane mechanism of electric stimulation remained incompletely understood. The necessity of bilateral ANT stimulation was affirmed in a pilocarpine model of secondarily generalized seizures in rats. Whereas unilateral anterior nucleus thalamotomy elicited no effect on pilocarpine-induced propensity or latency of developing seizures and status epilepticus, bilateral ANT stimulation significantly delayed the time to status epilepticus [22].

### ANT stimulation in drug-resistant epilepsy: efficacy and safety in the pre-SANTE era

The first clinical case series with thalamic lesioning for the control of epilepsy date back to 1967 [55]. Due to its involvement in seizure propagating circuitry (corticothalamic, mammillary, and the Papez circuits) Cooper and Upton, hypothesized in 1985 that “stimulation of the anterior nucleus of the thalamus should produce suppression of abnormal neural discharge within the limbic system” [79, 80]. In 1987, they described the bilateral ANT stimulation in six patients with drug-resistant complex partial seizures, which resulted in significant clinical control of the seizures in four of these patients. Subsequent to Cooper and Upton, several studies reported on the efficacy of bilateral ANT-DBS in drug-resistant epilepsy patients. The studies published in the pre-Sante era are summarized in Table 1 [27, 31, 39, 43, 60].

**Table 1:** Pre-SANTE clinical studies showing the efficacy of ANT-DBS in intractable epilepsy. *GTCS* generalized tonic-clonic seizures, *CPS* complex partial seizures, *DA* drop attacks, *SGTC* secondary generalized tonic-clonic seizures, *SPS* simple partial seizures, *TS* tonic seizures, *HMS* hypermotor seizures, *AMS* automotor seizures. Also reviewed in [32]

Authors, year	*N*	Seizure type	Stimulation parameters	Follow-up (months)	Mean seizure (%) reduction at last follow-up
Cooper et al., 1987	6	CPS	60–70 Hz, 3.5–3.8 V, 300 μs	42	n/a
Hodaie et al., 2002	5	GTCS, CPS, DA, CPS, SGTC	100 Hz, 10 V, 90 μs, 1 min on/5 min off, alternating left and right sides	12–21	54% (24–89%)
Kerrigan et al., 2004	5	SPS, CPS, SGTC	100 Hz, 1–10 V, 90 μs, 1 min on/10 min off, 5 min offset	6–36	48% (57–98%)
Osorio et al., 2007	4	CPS, SGTC, DA, SPS	175 Hz, 4.1 V, 90 μs, 1 min on/5 min off	36	75.6% (53–92%)
Lee et al., 2006	3	TS, DA, HMS, AMS, SGTCS	130 Hz, 1.5–7 V 90 μs, 1 min on/5 min off, alternating left and right sides	2–30	75.4% (50–90.6%)
Lim et al., 2007	4	GTCS,CPS, SPS SGTCS	90–110 Hz, 4–5 V, 60–90 μs, continuous	33–48	49% (35–76%)

The pre-SANTE studies show a variable treatment efficacy, which may be explained by the significant differences between the studies, including seizure type, follow-up and ANT-DBS stimulation parameters. Regarding the latter, initial stimulation parameters were based on experimental evidence, experience with stimulation of the central median nucleus of the thalamus for epilepsy and STN-DBS for Parkinson’s disease [43, 60]. All studies collectively concluded that ANT-DBS is a safe and well tolerable procedure, with minimal adverse events. Only one study reported a case of wound infection requiring system removal. Similar to the experience of DBS in movement disorders, the authors discuss a microthalamotomy effect, defined as a reduction in or abolition of symptoms with insertion of DBS alone. Hodaie et al. observed no additional seizure reduction after stimulation initiation and no increase in seizure frequency after stimulation cessation. In contrast, Kerrigan et al. report on an acute exacerbation of seizure frequency after discontinuation of stimulation, reversed by resuming stimulation. Osorio et al. did not observe the microthalamotomy effect.

## SANTE trial

These encouraging results culminated with the publication of a randomized double-blind controlled trial of Stimulation of the Anterior nuclei of Thalamus for Epilepsy (SANTE) which enrolled 110 patients with localization-related epilepsy [16]. One month after bilateral ANT implantation, patients were randomly assigned to a regime of stimulation (*n* = 54, 145 Hz, 5 V, 90 μs, 1 min on/5 min off) or no stimulation (*n* = 55, 0 V). After the 3 months blinded phase, all patients received stimulation. At month 13, all patients entered long-term follow-up in which stimulation parameters and AEDs varied freely. The long-term (5 years) efficacy and safety of this trial was reported in 2015 [64].

### Efficacy

At the end of the blinded phase, the stimulation group showed a relative greater estimated reduction of seizure frequency compared to the non-stimulated group with a difference of 29% (*p* = 0.0023). Secondary outcome measures: 50% responder rate, Liverpool Seizure Severity Scale (LSSS), and the Quality of life in Epilepsy (QoLIE-31) did not significantly differ at the end of the 3-month blinded phase. However, compared to baseline, all measures showed significant improvement at the end of the unblinded phase. At month 13 and 25, the median seizure frequency reduction was 41 and 56%, respectively, with corresponding 50% responder rates of 43, 54, and 67% at 37 months. Self-reported seizure severity decreased by 40% in the stimulated group compared to 20% in the control group (*p* = 0.047). Both LSSS and QoLIE-31 significantly improved at 13 and 25 months [16]. Long-term follow-up at 5 years showed a gradual increase of the mean percentage seizure reduction to 69%, with 11 participants reporting seizure freedom for at least 6 months. The 50% responder rates improved to 68% at 5 years [64]. The authors convincingly refute the confounding effect of discontinuation in the trial, due to poor response on improved outcome in terms of seizure reduction. However, the increased response rate may be influenced by these drop-outs during the 5-year follow-up period. In addition, the gradual prolonged increase (1–5 years) of the beneficial effect of ANT-DBS may also be influenced by additional AED regiment changes, tailoring of stimulation parameters and/or progressive improvement with stimulation [2, 64].

### Safety and tolerability

Reported adverse events (AE) at any time after implantation were most commonly hardware related (22.7%) consisting of paresthesia (18.2%), implant site pain (23.6%), implant site infection (12.7%), and electrode misplacement (8.2%). Procedural related AE such as intracerebral hematoma occurred in 4.5% of the patients, none of which were symptomatic.

There were no observed deaths during the operative month or 3-month double-blind phase. In total there were seven deaths during the study: one due to suicide, two definite, and two possible SUDEP, one due to cardiorespiratory arrest, and one died from liver cancer. None of which were considered to be device related by the authors.

Of the 105 participants entering the long-term follow-up, 30 discontinuations were reported (including six deaths, one before device implantation). Of these, 14 comprised device explants (implant site infection (2), device ineffectiveness (7), neuropsychological disorder (3), meningitis (1), and an undesirable change in stimulation (1)).

Although neuropsychological test scores for mood and cognition did not differ between the control and stimulated groups at the end of the blinded phase, significantly more patients in the stimulated group reported on AE relating to depression (14.8%) and memory impairment (13%) compared to the control group (1.8%, 1.8%). Depression related symptoms were reported in 32.7% and memory impairment in 27.3% of the patients during long-term follow-up. At 5-year follow-up several components of the neuropsychological examination showed gradual improvement from baseline including attention, executive function, depression, tension/anxiety, total mood disturbance, and subjective cognitive function. This paradoxical outcome regarding self-reported depression related symptoms and objective neurobehavioral testing was recently addressed by an in-depth and long-term analysis [78]. During the 7-year open label period, patients with prolonged ANT stimulation showed no cognitive decline or worsening of depression scores. In contrary, higher scores in executive functions and attention were observed at 7 years [78].

## ANT stimulation in drug-resistant epilepsy: efficacy and safety in the post-SANTE era

In the years after the publication of the SANTE trial, several case series further reported on the efficacy and safety of ANT-DBS in drug-resistant epilepsy, presented in Table 2 [34, 40, 59]. A case series of Piacentino et al. who qualitatively describe a cohort of six individual ANT-DBS patients is not included in this table. However, they report on a mean seizure reduction rate of more than 50% in patients with temporal lobe epilepsy [61]. AE reported by these case series comprise of implant site infection (three) of which two required hardware removal, and severe stimulation induced agitation requiring stimulation cessation. In concordance with the SANTE trial, one patient reported an increase in seizure frequency of 200% compared to baseline following stimulation initiation. With regard to the responding patients, Krishna et al. describe three patterns of seizure control (1) sustained (> 50%) seizure frequency reduction without stimulation initiation (prolonged insertional effect) (2) immediate stimulation effect: an increase in seizure frequency reduction immediately associated with stimulation initiation and (3) delayed stimulation effect: a decrease in mean seizure frequency with continued stimulation after initial failure of seizure reduction. Of note, an insertional effect was observed in 56% of the patients. Interestingly, in a case series reporting on seizure outcome after battery depletion, one patient with ANT-DBS and 3 years of continuous stimulation did not show a change in seizure frequency 6 months after battery depletion, either implicating a prolonged insertional effect or definite epileptic network modulation, or reflecting the natural course of epilepsy [13]. Lee et al. only observe a prolonged stimulation effect, as their study design rules out the possibility of a prolonged insertional effect, in which short-term outcomes remarkably associated with long-term seizure control. Regarding long-term cognitive functioning, Oh et al. report on slight improvement on fluency tasks and delayed verbal memory.

**Table 2:** Post-SANTE clinical studies showing the efficacy of ANT-DBS in drug-resistant epilepsy. *CPS* complex partial seizures, *SGTC* secondary generalized tonic-clonic seizures, *DA* drop attacks, *MC* myoclonic, *GTCS* generalized tonic-clonic seizures

Authors, year	*N*	Seizure type	Stimulation parameters	Follow-up (months)	Mean seizure (%) reduction at last follow-up
Krishna et al., *2015*	16	CPS, SGTC, DA, SPS, GTSC	100–185 Hz, 2.4–7 V, 90 μs, 1 min on/5 min off	51.6	11.5% (− 400–99%)*
Lee et al., *2012*	15	CPS, GTCS	100–185 Hz, 1.5–3.1 V, 90-150 μs, continuous	24–67	70.51 (0–100%)
Oh et al., *2011*	9	CPS, SGTC	100–185 Hz, 1.5–3.1 V, 90–150 μs, continuous	22–60	57.9% (35.6–90.4%)

*Median decrease seizure frequency for the whole cohort (*n* = 16)

## Treatment response

Although the efficacy and safety of ANT-DBS in drug-resistant epilepsy patients was convincingly shown in the SANTE trial, questions remain about the variability of responsiveness to treatment. In addition, two patients displayed a paradoxical response to ANT-DBS: one patient in the SANTE trial suffered from 210 brief partial seizures corresponding to the on-off cycle of stimulation in the blinded phase and one patient had a 200% increase of seizure frequency reported by Krisna et al. The variability of the treatment effect of ANT-DBS may be partially explained by the localization of the seizure onset zone, as patients with a seizure origin in one or both temporal lobes showed a greater response to ANT stimulation when compared to extratemporal, or multiple seizure onset-zones [16, 60]. Another explanation may be sought in the influence of the anatomical position of the active electrode on clinical outcome, as this could generate differential activation patterns by preferential stimulation of different subnuclei. In the SANTE trial, the DBS electrodes were placed presumably using a direct targeting method, therefore solely relying on its relative anatomical position of the ANT within the thalamus, and comparisons to the Schaltenbrand and Wahren atlas (SWA). The position of the active electrode within the ANT was verified visually on a post-operative magnetic resonance imaging (MRI). The role of micro-electrode recording (MER) in targeting the ANT and improving clinical response is unknown and not routinely applied. Interestingly, although not found to be clinically relevant, a post-hoc analyses of the SANTE study participants revealed that almost 10% of the electrodes were not within the limits of the ANT [21] via [91].

Recent proposed 3T MRI short tau inversion recovery and 1.5T T1 weighted magnetization prepared gradient echo (MPRAGE) images allow for visual delineation of the ANT. The imaging protocols are capable of clearly visualizing the anatomical boundaries of the ANT (mammillothalamic tract and the external medullary lamina) [29, 54]. As these imaging protocols were unavailable at the start of SANTE trial, this may provoke uncertainty about the exact location of the active electrodes. Particularly when considering the significant volumetrical and microstructural changes of the thalamus associated with increasing age; especially the anterior thalamus including the anterior-ventroanterior and dorsomedial nucleus [28]. In addition, ipsilateral thalamic atrophy has been demonstrated in patients with temporal lobe epilepsy but not in extratemporal and idiopathic generalized epilepsy [57]. Patients with mesial temporal lobe epilepsy show specific atrophy of the ANT, medial dorsal nucleus, and the medial pulvinar nucleus, with a concomitant decrease of thalamohippocampal connected volume [4]. Recent cohorts of ANT-DBS patients revealed a better clinical response when the active electrode was located within the anterior aspect of the ANT [34, 41]. Furthermore, non-responding to responding conversion was observed in four out five patients after re-programming the IPG to activate the most cranial contact [41]. Clear pre-operative visualization of the ANT therefore is likely to reduce the variability of responsiveness and may therefore further increase treatment effectiveness. Another cause of treatment variability may be sought in defining the optimal trajectory to the ANT. A transventricular approach is more susceptible to lead misplacement due to penetration of the lateral ventricles. However, other neurosurgeons advocate a transventricular approach as they observe an increased feasibility in reaching the ANT and less hardware related events [41].

## Mechanism of action

Despite its widespread clinical use, the exact mechanism of action of electrical stimulation on the central nervous system remains poorly understood. Initial hypothesis about the mechanism of DBS were based on the similarity between ablative procedures and high frequency stimulation with regard to treatment effect. High-frequency stimulation was therefore thought to function as a reversible lesion by inhibiting neurons near the stimulating electrode [8, 38, 63]. However, progressive understanding revealed that electrical fields have differential effects on different neuronal structures [26]. High-frequency thalamic DBS results in regions of both activation (axonal, within 2 mm of the electrode) and suppression (subthreshold, more than 2 mm of the electrode), where activation generates axonal output at the stimulus frequency [46]. Consequently, DBS may override or “hijack” the neural circuitry by blocking pathological activity and replacing efferent output [38]. Further evidence that ANT-DBS induces network modulation rather than simply inducing a local functional lesion arose from EEG and fMRI data. Indeed, ANT-DBS results in a pattern of cortical activation corresponding to the hodology of the ANT and therefore includes the Papez circuitry. Furthermore, the differential distribution of cortical activation is hypothesized to be dependent on the relative anatomical location of the active electrode within the ANT. Of note, cortical activation patterns are strongly dependent on stimulation amplitude and susceptible for considerable inter-and intra-individual variation [19, 95].

Other mechanisms by which ANT-DBS may remotely modulate neuronal network excitability is through local molecular hippocampal alterations. Unilateral ANT stimulation in kainic acid (KA) induced seizures in rats provoked decreased levels of glutamate and aspartate and an increase of GABA concentration in the ipsilateral CA3 region of the hippocampus [44]. This phenomenon has also been observed in the hippocampi of rhesus monkeys with mesial temporal lobe epilepsy induced by KA, indicating that ANT-DBS remotely inhibits KA-induced excitatory hyperactivation [66]. Secondly, chronic ANT-DBS may exert protective effects on hippocampal neurons and enhance the regeneration of neuronal fibers [48, 93]. Hippocampal neuronal cell apoptosis has been correlated with seizure frequency as was found in resected sclerotic hippocampi in patients with mesial temporal lobe epilepsy [92]. Vice versa, the number of neuronal cells negatively correlates with seizure frequency [45]. Although controversial, approaches to reduce neuronal cell loss may decrease seizure frequency [56, 94]. ANT-DBS has been shown to increase neurogenesis in the chronic stage of ANT-DBS in KA-induced seizures in rats as shown by an increased expression of Ki-67 and DCX [10]. The model of prolonged neurogenesis could further explain the observation of an increased efficacy of ANT-DBS in the long-term follow-up of the SANTE trial. Lastly, ANT-DBS may further induce neuroprotective effects by reversing the hippocampal pro-inflammatory state. In KA-induced seizures in rats, ANT-DBS induced a normalized gene expression of pro-inflammatory cytokines such as IL-1β and IL-6 and therefore prevent subsequent neuronal injury in the hippocampal CA1 [11] [1]. The involvement of inflammatory mediators in seizure susceptibility and epileptogenesis has been extensively recognized [67, 87].

Another mechanism which may underlie the therapeutic effect of ANT-DBS is through influencing glucose metabolism. In patients with temporal and frontal lobe epilepsy, an ipsilateral thalamic and hippocampal interictal glucose hypometabolism is often observed on FDG-PET, and its severity is correlated with a prolonged course of epilepsy [3, 6, 76]. Interestingly, a mouse model of chronic inhibition of brain energy metabolism showed that epileptiform activity could be induced by intracerebroventricular injection of a non-metabolizable glucose analog [65]. Strikingly, bilateral ANT stimulation promotes energy metabolism in the anterior thalamic region, thalamus and the hippocampus as measured by FDG-PET in rats [18]. Local ANT stimulation, induced increased glucose metabolism and may therefore reverse the predisposing thalamic hypometabolism and attenuate its deterioration. Further, ANT-DBS inhibits energy metabolism in the cingulate cortex and the frontal cortex [18]. The ANT-DBS induced hypometabolism was the most prominent in the motor cortex, which therefore through inhibition may increase the seizure threshold and thereby directly contribute to the anti-epileptic action of ANT-DBS. In contrast, bilateral ANT chemical lesioning did not show an increased glucose uptake in the bilateral anterior thalamic region nor did it induce neuronal energy metabolism changes in distant brain areas.

## ANT-DBS within the spectrum of neuromodulation for epilepsy

In addition to the ANT, several other brain structures have been targeted with stimulation for epilepsy and have been addressed in randomized controlled trials (RCTs) [17, 24, 33, 47, 74, 81–84, 90]. The intracranial targets include the centromedian thalamic nucleus, cerebellar cortex, hippocampus, nucleus accumbens, and responsive ictal onset zone stimulation. These RCTs have been systematically reviewed in a recently updated Cochrane meta-analysis [71]. In short, in addition to ANT-DBS (mean difference (MD): − 17.4% compared to sham stimulation), a statistical significant reduction in seizure frequency was found for responsive ictal onset zone stimulation (− 24%; multi-focal epilepsy) and hippocampal DBS (− 28.1%; temporal lobe epilepsy), with comparable adverse events in terms of frequency and severity [71]. However, no statistical significance was provided in terms of seizure freedom, responder rate or quality of life [71].

To date, there are no trials comparing intracranial stimulation to either lesser invasive modalities such as vagus nerve stimulation (VNS), transcutaneous-VNS (t-VNS) and repetitive transcranial magnetic stimulation (rTMS) or best medical practice. Two RCTs report on a similar or slightly inferior treatment response for VNS (MD − 12.7% and − 18.4%) when compared with intracranial targets [23, 62]. A direct comparison between VNS and ANT-DBS concerning treatment outcome should be made with caution as almost half (44.6%) of the SANTE study population received VNS implantation prior to ANT stimulation [16]. With regard to rTMS, a recent Cochrane review concluded that, although reasonable evidence suggests that rTMS is effective at reducing epileptiform discharges, strong evidence is lacking for the efficacy of rTMS for seizure reductions in drug-resistant epilepsy [9]. Furthermore, add-on therapy with t-VNS (25 Hz) has not been proven superior compared to active controls (1 Hz) after 20 weeks in patients with drug-resistant epilepsy [5].

## Concluding remarks and future directions

ANT-DBS for drug-resistant epilepsy is a safe and well-tolerated therapy, where a particular emphasis must be given to monitoring of depression and memory function. ANT-DBS is an efficacious treatment modality, even when curative procedures or lesser invasive neuromodulative techniques failed. When compared to VNS, ANT-DBS shows slightly superior treatment response, which urges for direct comparative trials. Based on the available evidence ANT-DBS and VNS therapies are currently both superior compared to non-invasive neuromodulation techniques such as t-VNS and rTMS. Despite its clinical efficacy, ANT-DBS for drug-resistant epilepsy still faces great challenges. Optimization of the procedural DBS protocol including imaging techniques, surgical procedure, and algorithms for adaptation of stimulation parameters could aid to reduce the treatment response variability. Additional research will have to provide for better understanding of normal physiological neuronal networks compared to epileptogenic networks in order to gain more insight into the mechanism of action ANT-DBS in localization-related epilepsy. Ideally, further in-depth knowledge of epileptogenic networks may explain for the differential response of DBS of different anatomical targets in different seizure types [16, 17, 42, 68, 69, 85]. Furthermore, adaptive (seizure-dependent) ANT-DBS may increase the efficacy, efficiency and selectivity of this treatment as is observed in focal responsive cortical stimulation and subthalamic nucleus DBS for Parkinson’s disease [7, 77]. Randomized clinical studies in search for the optimal target in well-defined epilepsy patient populations will ultimately allow for optimal patient stratification when applying intracranial neuromodulation therapy for drug-resistant epilepsy patients.
